# Changes in Sarcopenia and Incident Depression in Prospective Cohorts

**DOI:** 10.3390/jcm15114015

**Published:** 2026-05-22

**Authors:** Furong Qu, Qingyue Zeng, Yuan Yao, Jiaxi Zhao, Zhipeng Li

**Affiliations:** General Practice Ward/International Medical Center Ward, General Practice Medical Center, West China Hospital, Sichuan University, Chengdu 610041, China; qufurong66@wchscu.cn (F.Q.); 2024324025096@stu.scu.edu.cn (Q.Z.); yaoyuan@wchscu.cn (Y.Y.);

**Keywords:** sarcopenia, depression, dynamic nature, epidemiology

## Abstract

**Background**: Previous studies have confirmed an association between sarcopenia and depression but were mostly based on single baseline assessments, ignoring dynamic changes in sarcopenia status. This study aimed to investigate the association between changes in sarcopenia status and incident depression risk. **Methods**: Data were sourced from the China Health and Retirement Longitudinal Study (CHARLS). Sarcopenia status was classified as non-sarcopenia, possible sarcopenia, or sarcopenia according to AWGS 2019 criteria. Changes in status were defined by comparing assessments at baseline (2011) and two years later (2013). Depression was assessed using the CESD-10 (score ≥10). Cox proportional hazards models were used to calculate hazard ratios (HRs) and 95% confidence intervals (CIs). **Results**: Participants who progressed from non-sarcopenia to possible sarcopenia or sarcopenia had a higher risk of depression than those who remained non-sarcopenic (HR 1.30, 95% CI 1.14–1.47). Conversely, participants who recovered from sarcopenia had a lower risk of depression than those with persistently stable sarcopenia (HR 0.65, 95% CI 0.45–0.94). **Conclusions**: Changes in sarcopenia status are differentially associated with the risk of incident depression. The progression of sarcopenia status is associated with an increased risk of incident depression, whereas recovery from sarcopenia is linked to a reduced risk.

## 1. Introduction

Sarcopenia is a geriatric syndrome characterized by progressive declines in skeletal muscle mass, strength, and physical function, with prevalence ranging from 1% to 29% in community-dwelling older adults and up to 33% in long-term care institutions [1]. Notably, this wide variation largely reflects differences in diagnostic criteria, measurement tools, study population characteristics, and the selection of cut-off values. Sarcopenia not only leads to fractures, limited mobility, and reduced quality of life but also frequently coexists with cardiovascular disease and metabolic disorders [2,3,4,5]. Depression is also highly prevalent in older adults and is significantly associated with sarcopenia. Both conditions share modifiable risk factors such as smoking and physical inactivity [6,7] and may be linked by a bidirectional vicious cycle, as depression itself is a known risk factor for reduced physical activity and poor nutrition, both of which can accelerate muscle loss [8].

From a biological perspective, chronic inflammation associated with sarcopenia can trigger neuroinflammation, interfere with monoamine neurotransmitter metabolism, and reduce the expression of brain-derived neurotrophic factor, all of which are key elements in the pathophysiology of depression [9,10]. Second, hypothalamic–pituitary–adrenal axis dysregulation leads to elevated cortisol levels, promoting muscle catabolism and impairing mood regulation [11]. Third, myokines (e.g., irisin) released during muscle contraction can cross the blood–brain barrier, promote hippocampal neurogenesis and synaptic plasticity, and exert antidepressant effects. Sarcopenia may reduce myokine secretion, potentially increasing vulnerability to depression [12,13].

Previous cross-sectional or single-baseline studies have repeatedly shown a link between sarcopenia and depression [14,15,16]. Yet, muscle health is not static; lifestyle modifications and medical interventions can lead to clinically meaningful improvement or deterioration over relatively short periods [17,18]. Consequently, inferences drawn from a single time point cannot fully characterize the temporal interplay between sarcopenia and depression, leaving a gap in evidence-based guidance for monitoring and intervention strategies.

To address this gap, the present study leveraged the large sample, multi-time point measurements, and long-term follow-up data of the nationally representative CHARLS, along with its repeated assessments of sarcopenia parameters and long-term tracking of depression outcomes, to investigate the longitudinal association between dynamic transitions in sarcopenia status and the risk of incident depression among the middle-aged and older Chinese population [19].

## 2. Methods

### 2.1. Study Design and Population

**Figure 1:**
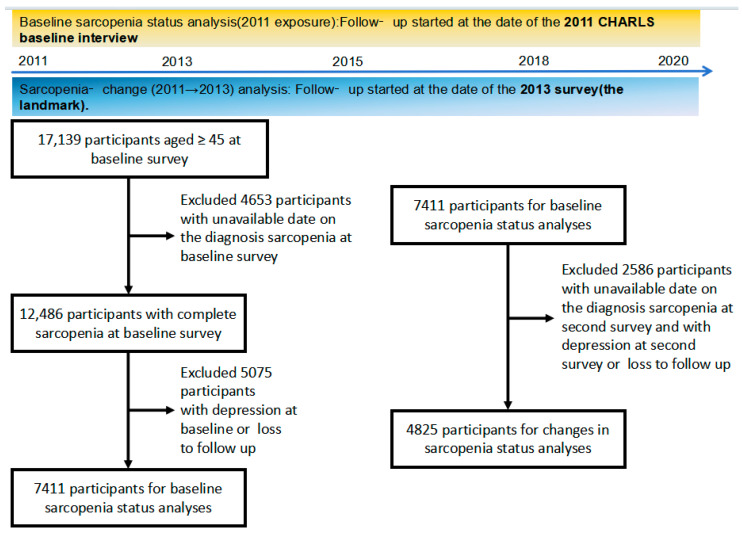
Selection process for the study population.

Data were sourced from the China Health and Retirement Longitudinal Study (CHARLS), a nationwide prospective cohort [19]. CHARLS used a multistage stratified probability sampling design to recruit a nationally representative sample of Chinese residents aged ≥45 years. Further details are provided in the Supplementary Methods. Detailed information about the study design is summarized in the “Methods” section of the Supplementary Data. The study follows a structured timeline with multiple survey waves, allowing for comprehensive tracking of participants’ health and demographic changes over time. The baseline survey, conducted in 2011 (Wave 1), established initial health and demographic profiles of the participants, providing a foundation for longitudinal analysis. In 2013 (Wave 2), a follow-up survey was conducted to assess dynamic changes in sarcopenia status. Subsequent follow-up waves continued through 2020 (Wave 5), enabling researchers to monitor long-term health outcomes, including the onset of depression. CHARLS received ethical approval from the Institutional Review Board of Peking University (IRB approval code: IRB 00001052-11014). As CHARLS is a longitudinal social survey and not a clinical trial, clinical trial registration is not applicable. Informed consent was obtained from all participants [20]. For the longitudinal analysis of sarcopenia change and incident depression, we used a landmark design with the 2013 CHARLS survey as time zero. Participants were required to be free of depression at both the 2011 baseline and the 2013 survey. Follow-up time was calculated from the 2013 interview date until the first occurrence of depression, death, loss to follow-up, or the end of available data. This approach inherently excludes incident depression events that occurred between 2011 and 2013 and eliminates immortal time bias. This study employed a transition-based analysis rather than a time-varying exposure model, as exposure status (changes in sarcopenia status between Wave 1 and Wave 2) was defined based on the comparison between two fixed time points. To assess potential selection bias, we compared baseline characteristics among the overall CHARLS population (*n* = 17,139), the baseline analytical cohort (*n* = 7411), and the longitudinal transition cohort (*n* = 4825). The results are presented in Supplementary Table S16. Figure 1 illustrates the process of study population selection. Of the 17,139 participants in CHARLS, a total of 4653 participants were excluded due to missing baseline sarcopenia data, which included the absence of key measurements such as skeletal muscle mass (ASM), handgrip strength, and physical function. Additionally, 5075 participants were excluded due to missing depression data, baseline depression, or loss to follow-up. Following the implementation of strict exclusion protocols, the baseline cross-sectional analysis incorporated 7411 individuals. The longitudinal evaluation of muscle status transitions further excluded 2586 subjects, yielding a final analytical cohort of 4825 participants.

### 2.2. Assessment of Sarcopenia Status

The 2019 AWGS framework was adopted to classify sarcopenia, requiring the assessment of muscle strength, ASM, and physical function [21]. Based on this framework, participants were categorized into three mutually exclusive groups: non-sarcopenia, possible sarcopenia, and sarcopenia. Sarcopenia is diagnosed when low muscle mass occurs with either low strength or poor function. Possible sarcopenia is defined as low strength or poor function alone.

Muscle strength was measured using a Yuejian TM WL-1000 dynamometer (Yuejian, Shanghai, China), with low grip strength defined as <28 kg for men and <18 kg for women [19]. Anthropometric measurements (height and weight) were obtained using standardized digital scales and stadiometers (manufactured in China; accurate to 0.1 kg and 0.1 cm, respectively) [22]. The ASM was estimated using a validated anthropometric equation for Chinese residents, which has shown strong agreement with dual X-ray absorptiometry (DXA) [23,24]. Low muscle mass was defined as the sex-specific lowest 20% of height-adjusted ASM (ASM/Ht^2^) in the study population [23,25]. Because CHARLS did not collect DXA-measured ASM, we used these population-specific cut-offs, as recommended for studies without direct DXA measurements [26,27]. The sex-specific ASM/Ht^2^ cut-off values were <7.01 kg/m^2^ for men and <5.31 kg/m^2^ for women in 2011, <7.05 kg/m^2^ for men and <5.38 kg/m^2^ for women in 2013, and <7.07 kg/m^2^ for men and <5.39 kg/m^2^ for women in 2015. Notably, these values closely aligned with the AWGS 2019 recommended cut-offs (<7.0 kg/m^2^ for men and <5.4–5.7 kg/m^2^ for women), supporting the validity of our approach.

Physical performance was assessed using gait speed and the chair stand test, following the method described by Wu et al. [22]. Low physical performance was defined as a gait speed <1.0 m/s or a time of ≥12 s to complete five chair stands, consistent with AWGS 2019 criteria. Further details about the definitions of sarcopenia components in the CHARLS have been described previously [21]. Changes in sarcopenia status between 2011 and 2013 were categorized as: (1) stable non-sarcopenia, (2) progression (non-sarcopenia to possible sarcopenia or sarcopenia), (3) stable possible sarcopenia, (4) recovery from possible sarcopenia (to non-sarcopenia), (5) progression from possible sarcopenia (to sarcopenia), (6) stable sarcopenia, and (7) recovery from sarcopenia (to non-sarcopenia or possible sarcopenia).

### 2.3. Ascertainment of Covariates

Our analytical models accounted for a comprehensive array of confounders: age, biological sex, marital status (married vs. other), educational attainment (middle school/below vs. high school/above), residence (urban vs. rural), smoking and alcohol habits, physical functionality, and BMI. Clinical biomarkers and comorbidities included systolic blood pressure (SBP), C-reactive protein (CRP), glycated hemoglobin (HbA1c), triglycerides (TG), high-density lipoprotein cholesterol (HDL-C), diabetes, hypertension, and dyslipidemia. Activities of daily living (ADL) function was assessed using a 6-item activities of daily living scale, evaluating difficulties in dressing, bathing, eating, bed transfers, toileting, and continence. Scores ranged from 0 to 6, with 0 indicating no impairment and ≥1 indicating impairment.

### 2.4. Assessment of Depression Events

Depression was assessed using the 10-item Center for Epidemiologic Studies Depression Scale (CESD-10), a validated screening tool for Chinese older adults, with a cut-off score of ≥10 defining possible depression (references) [28,29]. Previous research has shown that the CESD-10 is a reliable depression screening tool, with good internal consistency, sensitivity, and specificity [30]. It has also been validated for use in Chinese older adults [31,32]. The scale assesses psychological well-being over the past week, using 10 questions like “I felt depressed” and “I was happy.” Responses are rated on a 0 to 3 scale, with two positive questions scored in reverse. Detailed descriptions can be found in previous publications [33]. The total score ranges from 0 to 30, and a score of 10 or higher indicates possible depression [34].

### 2.5. Statistical Analysis

Continuous data were summarized as means ± SD or medians [IQR], and categorical variables as frequencies. We used Cox proportional hazards regression to evaluate the association between sarcopenia status (baseline or change) and incident depression, with follow-up time calculated from the respective baseline (2011 for baseline analysis; 2013 for change analysis). Hazard ratios (HRs) and 95% confidence intervals (CIs) were estimated using four sequentially adjusted models: Model 0 (unadjusted); Model 1 (age + sex); Model 2 (marital status, residence, education); and Model 3 (full adjustment for lifestyle factors, physical function, BMI, blood pressure, metabolic biomarkers, and chronic disease history, as listed in Table 1). For missing data, Supplementary Table S1A,B summarize missing rates for covariates. Missing values were handled using multiple imputation by chained equations (MICE) (detailed in the Supplementary Materials). In the CHARLS, we only imputed the covariates in which the missing rates were less than 80% recommended by previous studies) [35,36], in each cohort, we performed 5 imputations and generated 5 imputed datasets. Effect estimates were calculated separately for each of the 5 datasets and then pooled according to Rubin’s rules [37]. Convergence of the imputation procedure was assessed by inspecting trace plots and calculating the potential scale reduction factor (Gelman-Rubin diagnostic) for each imputed variable; all values were ≤1.05, indicating adequate convergence. We assumed missingness of exposure and outcome data to be missing at random (MAR) conditional on observed covariates.

A stratified analysis was performed by sex and age group (middle-aged <65 years, older adults ≥65 years) to explore the impact of sarcopenia status changes on depression risk. Likelihood ratio tests were used to assess statistical interactions. Sensitivity analyses were conducted to validate the robustness of findings, including (i) re-evaluating sarcopenia status in the third-wave survey (Supplementary Figure S2), (ii) separately analyzing the effects of “possible sarcopenia vs. non-sarcopenia” and “sarcopenia vs. possible sarcopenia” groups, (iii) additionally adjusting for the use of antihypertensive and antidiabetic medications.

All statistical analyses were conducted using R version 4.4.1. A two-sided *p*-value of *p* < 0.05 was considered statistically significant.

## 3. Results

### 3.1. Baseline Characteristics of the Study Population

The baseline cohort (*n* = 7411; 46.4% female; mean age 58.3 years) revealed distinct demographic patterns (Table 1). Compared to non-sarcopenic peers, affected individuals tended to be older, unmarried, less educated, and rural residents. Clinically, they exhibited lower BMI, HbA1c, and TG, alongside elevated CRP and HDL-C levels.

For the analysis of changes in sarcopenia status, 4825 participants (46.7% female, mean age 58.2 years) were included according to the relevant criteria, and their baseline characteristics are presented in Table S2. Baseline characteristics of participants without imputation are also described in Supplementary Tables S3 and S4, showing similar results to those in Table 1 and Table S2. To assess potential selection bias, we compared the baseline characteristics of the overall CHARLS population (*n* = 17,139), the baseline analytical cohort (*n* = 7411), and the longitudinal transition cohort (*n* = 4825). As shown in Supplementary Table S16, participants excluded due to missing data were slightly older and had a higher proportion of females, but the overall distributions of key variables were largely comparable.

During a median follow-up of 9 years, 3136 cases of incident depression were recorded in the baseline cohort. In the longitudinal transition cohort (*n* = 4825; median follow-up 7 years), 2217 participants developed depression.

### 3.2. Association of Baseline Sarcopenia Status with Incident Depression

**Figure 2:**
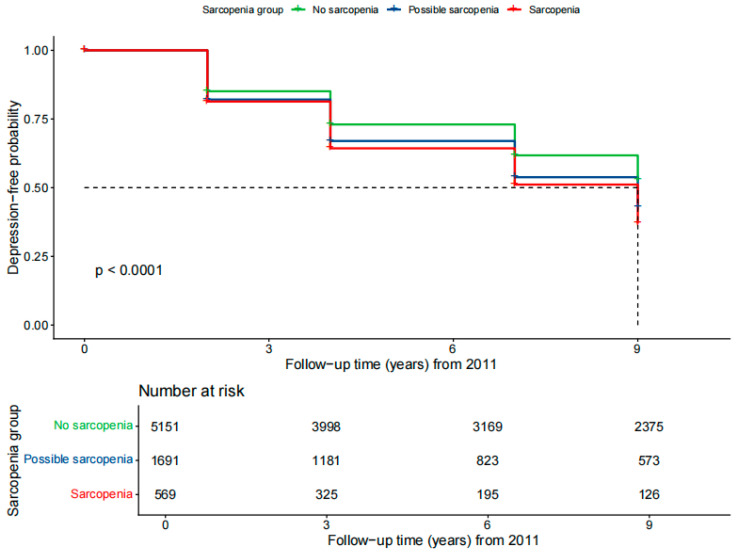
Kaplan–Meier curves for incident depression by baseline sarcopenia status. The curves show depression-free survival probability from 2011 to 2020 for participants with non-sarcopenia (*n* = 5151), possible sarcopenia (*n* = 1691), and sarcopenia (*n* = 569) at baseline. Log-rank test *p* < 0.0001.

The relationship between sarcopenia at baseline and incident depression is summarized in Supplementary Table S5. Multivariable-adjusted analysis revealed that sarcopenia was associated with a 25% increased risk of depression (HR 1.25, 95% CI 1.08–1.46), while possible sarcopenia carried a 22% higher risk (HR 1.22, 95% CI 1.13–1.33). The incidence rates of depression per 1000 person-years were 66.54, 85.13, and 95.04 for participants with non-sarcopenia, possible sarcopenia, and sarcopenia at baseline, respectively (Supplementary Table S14). The Kaplan–Meier survival curves for participants with different baseline sarcopenia status are shown in Figure 2. The log-rank test indicated a significant difference in depression-free survival across groups (*p* < 0.0001). The depression-free survival probability was highest among participants with non-sarcopenia, intermediate in those with possible sarcopenia, and lowest in those with sarcopenia, with the curves diverging progressively over time (log-rank *p* < 0.0001). The proportional hazards assumption was tested using Schoenfeld residuals; no significant violations were detected (global test *p* = 0.18), indicating that the assumption was met.

### 3.3. Association of Changes in Sarcopenia Status with Incident Depression

During the two-year follow-up, 18.7% (*n* = 649) of participants who were initially non-sarcopenic progressed to either possible sarcopenia or sarcopenia. Conversely, 58.9% (*n* = 174) of those with baseline sarcopenia experienced recovery to a better status (Table 2). Table 3 illustrates the relationship between these transitions and depression risk. Compared to individuals who remained non-sarcopenic, those who deteriorated from a non-sarcopenic state to possible sarcopenia or sarcopenia exhibited a 30% higher risk of developing depression (HR 1.30, 95% CI 1.14–1.47). In contrast, among those initially diagnosed with sarcopenia, improvement to a non-sarcopenic or possible state was associated with a protective effect (HR 0.65, 95% CI 0.45–0.94). For the cohort with possible sarcopenia at baseline, returning to a non-sarcopenic state was associated with a reduced risk of depression (HR 0.75, 95% CI 0.63–0.91), whereas progression to sarcopenia did not yield a statistically significant change in risk (HR 1.25, 95% CI 0.74–2.11). The incidence rates of depression per 1000 person-years for each transition group are presented in Supplementary Table S15. Participants who progressed from non-sarcopenia to possible sarcopenia or sarcopenia had an incidence rate of 116.28 per 1000 person-years, compared with 86.59 for those who remained stable non-sarcopenic. Among those with baseline sarcopenia, recovery to non-sarcopenia or possible sarcopenia was associated with an incidence rate of 103.11 per 1000 person-years, whereas persistent sarcopenia had a rate of 169.68 per 1000 person-years. The Kaplan–Meier survival analysis for changes in sarcopenia status (Supplementary Figures S3–S5) showed that participants who progressed from non-sarcopenia to possible sarcopenia or sarcopenia had consistently lower depression-free survival than those who remained stable (log-rank *p* < 0.0001). Among those with baseline sarcopenia, recovery to non-sarcopenia or possible sarcopenia was associated with higher depression-free survival compared to persistent sarcopenia (log-rank *p* = 0.005). Similar patterns were observed for possible sarcopenia at baseline (log-rank *p* < 0.0001).

### 3.4. Subgroup Analyses and Sensitivity Analyses

In our subgroup investigations, a deteriorating sarcopenia status was a consistent predictor of incident depression, regardless of gender or age (all *p* < 0.05, Supplementary Tables S6–S8). However, the impact of status recovery varied: clinical improvement from sarcopenia was significantly linked to lower depression risk in female and older participants (≥65 years), but these associations did not reach statistical significance in the male and younger cohorts (Supplementary Table S8). Among those with possible sarcopenia at baseline, the risk reduction associated with recovery to a non-sarcopenic state was particularly pronounced in men (HR 0.53, 95% CI 0.40–0.69) and both age groups, yet remained non-significant in the female subgroup (Supplementary Table S7).

Sensitivity analyses using data from the third survey largely supported the primary findings for progression from non-sarcopenia and recovery from possible sarcopenia, but the protective association of sarcopenia recovery was attenuated and no longer statistically significant, likely due to reduced sample size (Supplementary Tables S9 and S10). When the groups were analyzed using separate classifications for possible sarcopenia and sarcopenia, the association between progression to possible sarcopenia and depression risk remained significant, while progression directly to sarcopenia showed a consistent direction but did not reach statistical significance (Supplementary Table S13). Furthermore, the observed risks associated with sarcopenia transitions also remained significant after adjusting for the potentially confounding effects of antidiabetic and antihypertensive therapies (Supplementary Tables S11 and S12).

## 4. Discussion

In this prospective cohort study, we examined the associations of baseline and changes in sarcopenia status with incident depression risks. Participants with possible sarcopenia and sarcopenia showed elevated risks of incident depression compared with non-sarcopenic participants. Moreover, non-sarcopenic participants who progressed to possible sarcopenia/sarcopenia status also showed elevated risks of incident depression compared with stable non-sarcopenic participants. However, patients with possible sarcopenia who recovered to non-sarcopenia status and sarcopenic participants who recovered to non-sarcopenia or possible sarcopenia status showed decreased incident depression risks when compared with their stable counterparts.

Our findings are consistent with a recent prospective study by Li et al., who used data from the Western China Health and Aging Trends (WCHAT) study and reported that baseline sarcopenia was associated with a 44% higher risk of incident depressive symptoms (HR 1.44, 95% CI 1.13–1.83) [16]. The slightly smaller effect size in our study (HR 1.30 for progression) may be explained by differences in outcome measurement (CESD-10 vs. GDS-15), sarcopenia definition (population-specific cut-offs vs. fixed AWGS cut-offs), and follow-up duration. Importantly, our study extends these findings by focusing on transitions in sarcopenia status rather than a single baseline assessment. We demonstrated that progression from non-sarcopenia to possible sarcopenia or sarcopenia was associated with a higher risk of depression, whereas recovery from sarcopenia was associated with a lower risk. Prior evidence [38] involving 4395 individuals and over 10,000 assessments has already illustrated the dynamic changes in this condition: 60.3% of that cohort stayed in the “possible sarcopenia” category, while 24.5% improved and 6.7% deteriorated. Our results align with this dynamic pattern within the CHARLS population. Notably, we observed a higher proportion of recovery from possible sarcopenia (58.6%) compared to the 24.5% reported by Luo et al. and a lower proportion of stable possible sarcopenia (38.3% vs. 60.3%) [39]. These discrepancies likely reflect differences in follow-up duration (we used a two-year transition window while Luo et al. examined changes across multiple waves) and cohort composition. Nonetheless, the overall pattern of sarcopenia as a dynamic rather than static condition is consistently observed across studies.

In the analysis above, we combined “possible sarcopenia” and “sarcopenia” into a single transition category. From a clinical perspective, both represent meaningful declines in muscle health—possible sarcopenia indicates reduced strength or physical performance, while sarcopenia additionally involves low muscle mass, and both are linked to adverse outcomes. From an analytical standpoint, transitions from non-sarcopenia to either category reflect worsening muscle health, and combining them allows us to assess the overall effect on depression risk while maintaining statistical power. Separate analyses for each category are presented in Supplementary Table S13. In this sensitivity analysis, we observed that progression from non-sarcopenia to possible sarcopenia was significantly associated with a higher risk of depression (HR 1.32, 95% CI 1.15–1.51), whereas direct progression to sarcopenia showed a similar direction but did not reach statistical significance (HR 1.20, 95% CI 0.92–1.56). This may be due to the relatively small number of participants who progressed directly to sarcopenia (*n* = 139) and the resulting limited statistical power, or it may suggest that the intermediate state of possible sarcopenia already captures the majority of the depression risk associated with muscle health decline. Nevertheless, the overall pattern across the analyses suggests that worsening sarcopenia status tends to be associated with an elevated risk of depression, although the precision of estimates for the most severe deterioration may be constrained by sample size.

We further assessed the robustness of our findings using data from the third survey. The results showed that, among participants with possible sarcopenia at baseline, those who progressed to sarcopenia exhibited an increased risk of depression, but this finding did not reach statistical significance, likely due to the small sample size in the group (*n* = 34) and the low number of depression cases (*n* = 16), resulting in limited statistical power [40]. Furthermore, alternative explanations should also be considered, including measurement variability in sarcopenia components across the two time points, potential misclassification of sarcopenia status due to the use of anthropometric equations rather than DXA, and the possibility that the transition from possible sarcopenia to sarcopenia represents a less clinically impactful change compared to the transition from non-sarcopenia to sarcopenia. Larger cohort studies are needed to clarify this specific transition.

In addition, our subgroup analyses revealed a clinically meaningful sex difference. Recovery from sarcopenia was associated with a significant reduction in depression risk in female participants but not in male participants (Table S8). This sex difference may be explained by biological, psychosocial, and behavioral factors. Biologically, estrogen has neuroprotective and anti-inflammatory effects; its decline after menopause may render women more susceptible to the mental health benefits of muscle recovery [41,42]. Psychosocially, women are more likely to engage in social physical activities during rehabilitation, which may confer additional mood benefits through social support [43,44]. Additionally, women generally have a higher likelihood of seeking healthcare, potentially leading to more effective sarcopenia management [45]. Notably, among participants with possible sarcopenia at baseline, recovery to non-sarcopenia status was associated with a particularly pronounced reduction in depression risk in men (HR 0.53), but the association was not significant in women. This contrasting pattern suggests that men may derive greater benefit from recovering from milder muscle function impairment (possible sarcopenia), whereas women may only exhibit a significant psychological benefit when they recover from more severe sarcopenia. This may be related to the tendency of men to actively engage in exercise to achieve recovery when their function is still relatively preserved, while women often seek intervention only when muscle health has already deteriorated. It should be emphasized that the subgroup sample sizes were limited, and some non-significant findings may be due to limited statistical power. Thus, the above interpretations require confirmation in future studies.

The findings of this study offer some insights for clinical practice and public health policy. Firstly, incorporating sarcopenia screening into comprehensive geriatric assessments for depression risk may hold potential value. In resource-limited or primary healthcare settings, the use of simple, low-cost screening tools is crucial. The SARC-F questionnaire, a self-reported scale consisting of five questions regarding strength, assistance with walking, rising from a chair, climbing stairs, and falls, has been validated for sarcopenia screening and can be applied in routine geriatric assessments without requiring specialized equipment [46,47]. Similarly, handgrip dynamometry represents an economical, portable, and rapid method of detection that can be widely promoted for use in community health centers or general practice clinics [21,48]. These tools help identify high-risk individuals, enabling them to benefit from further evaluation or preventive interventions even when dual-energy X-ray absorptiometry (DXA) or bioelectrical impedance analysis is unavailable. Secondly, evidence-based interventions aimed at delaying the progression of sarcopenia may effectively reduce the incidence of depression. For instance, resistance training (2–3 sessions per week) remains the cornerstone of sarcopenia management [49,50]. Beyond directly increasing muscle mass and strength, exercise exerts clear antidepressant effects through neuroendocrine regulation, promoting the expression of brain-derived neurotrophic factor (BDNF) and reducing systemic inflammation [51,52]. Additionally, adequate protein intake (1.2–1.5 g/kg body weight per day) along with supplementation with leucine-rich amino acids, vitamin D, and omega-3 fatty acids has been proven beneficial for maintaining muscle function [53,54]. These nutritional strategies, characterized by low risk and wide availability, can be incorporated into depression prevention programs for older adults. It is noteworthy that although our study demonstrates an association between sarcopenia remission and a reduced risk of depression, observational data cannot establish causality. Well-designed randomized controlled trials are urgently needed to clarify whether exercise, nutritional, or multimodal interventions targeting sarcopenia can effectively reduce the risk of new-onset depression in the elderly population.

This research possesses several merits. First, to the best of our knowledge, this study is among the first to longitudinally assess the relationship between transitions in sarcopenia status and the incidence of depression. Second, the data for this study are derived from a large, nationally representative prospective cohort study of middle-aged and older Chinese people, with its sample size and follow-up period providing sufficient statistical power. Third, we used repeated measurements of sarcopenia status (2011 and 2013) and a categorical definition of transitions (non-sarcopenia, possible sarcopenia, sarcopenia), which aligns with clinical diagnostic frameworks and enhances interpretability. Fourth, we have validated the robustness of our research conclusions through multiple sensitivity analyses, and the consistency of results across these analyses supports the reliability of our findings.

Several additional limitations warrant consideration. First, while we adjusted for a range of potential confounders, residual confounding from unmeasured factors (e.g., nutritional status, social support, dietary patterns, medication use, genetic predisposition) may persist. Second, the bidirectional nature of the sarcopenia-depression relationship cannot be fully addressed in our analytic design; although we temporally ordered exposure before outcome, depression symptoms at earlier time points could have contributed to muscle decline via reduced physical activity and poor nutrition. Third, the CESD-10 is a screening tool rather than a clinical diagnostic interview for major depressive disorder, which may lead to outcome misclassification. Fourth, using population-specific cut-offs (lowest 20%) may yield higher prevalence estimates of low muscle mass than fixed AWGS cut-offs, limiting comparability with other studies. However, our derived cut-off values were very close to AWGS thresholds, suggesting minimal practical impact. This choice is unlikely to substantially alter the direction or magnitude of the risk associations. Regarding risk associations, the choice of cut-offs primarily affects classification at the diagnostic boundary and is unlikely to substantially alter the hazard ratios. Thus, our main findings are robust to this methodological choice. Fifth, we did not perform a formal sensitivity analysis for attrition bias (e.g., inverse probability censoring weighting) because detailed reasons for loss to follow-up were not available in the CHARLS dataset. However, the baseline characteristics of our analytical cohorts were largely comparable to those of the overall CHARLS population (Supplementary Table S16). We reported total person-years and event/censoring counts (Supplementary Tables S14 and S15). While attrition bias cannot be completely ruled out, the consistency of our findings across multiple sensitivity analyses suggests that it is unlikely to have substantially affected the results. Additionally, our findings are derived from a Chinese cohort and may not be directly generalizable to other ethnic or geographic populations.

## 5. Conclusions

Changes in sarcopenia status are differentially associated with the risk of incident depression. The progression of sarcopenia status is associated with an increased risk of incident depression, whereas recovery from sarcopenia is linked to a reduced risk. Future research is needed to develop precise prevention strategies to delay sarcopenia progression as well as tailored interventions to reverse sarcopenia in depression management practice.

## Figures and Tables

**Table 1:** Baseline characteristics of participants for baseline sarcopenia status analyses.

Characteristics	Total (*n* = 7411)	Non-Sarcopenia (*n* = 5151)	Possible Sarcopenia (*n* = 1691)	Sarcopenia (*n* = 569)	*p* Value
Age, mean (SD), years	58.29 ± 9.11	56.32 ± 7.99	60.68 ± 9.22	69.03 ± 9.11	<0.0001
Sex, *n* (%)					<0.0001
Female	3435 (46.35)	2250 (43.68)	904 (53.46)	281 (49.38)	
Male	3976 (53.65)	2901 (56.32)	787 (46.54)	288 (50.62)	
Marital status, *n* (%)					<0.0001
Married	6720 (90.68)	4804 (93.26)	1491 (88.17)	425 (74.69)	
Others	691 (9.32)	347 (6.74)	200 (11.83)	144 (25.31)	
Education, *n* (%)					<0.0001
Junior and below	6334 (85.47)	4271 (82.92)	1521 (89.95)	542 (95.25)	
Senior and above	1077 (14.53)	880 (17.08)	170 (10.05)	27 (4.75)	
Residence, *n* (%)					<0.0001
Rural	4361 (58.84)	2967 (57.60)	978 (57.84)	416 (73.11)	
Urban	3050 (41.16)	2184 (42.40)	713 (42.16)	153 (26.89)	
Drinking status, *n* (%)					<0.0001
Ever drinkers	3274 (44.18)	2396 (46.52)	645 (38.14)	233 (40.95)	
Never drinkers	4137 (55.82)	2755 (53.48)	1046 (61.86)	336 (59.05)	
Smoking status, *n* (%)					<0.0001
Ever smokers	3195 (43.11)	2319 (45.02)	629 (37.20)	247 (43.41)	
Never smokers	4216 (56.89)	2832 (54.98)	1062 (62.80)	322 (56.59)	
BMI, mean (SD), kg/m^2^	23.76 ± 3.86	23.92 ± 3.79	24.85 ± 3.47	19.08 ± 1.66	<0.0001
Physical activity function					<0.0001
no	6985 (94.25)	4937 (95.85)	1553 (91.84)	495 (86.99)	
yes	426 (5.75)	214 (4.15)	138 (8.16)	74 (13.01)	
SBP, mean (SD), mmHg	130.60 ± 21.04	129.29 ± 19.92	133.81 ± 22.28	132.96 ± 25.44	<0.0001
CRP, mean (SD), mg/L	2.74 ± 7.59	2.55 ± 7.23	3.06 ± 8.05	3.51 ± 9.17	<0.01
HbA1c, mean (SD), %	5.26 ± 0.80	5.25 ± 0.76	5.32 ± 0.91	5.19 ± 0.69	<0.001
Triglycerides, mean (SD), mmol/L	1.55 ± 1.21	1.57 ± 1.29	1.56 ± 1.07	1.27 ± 0.78	<0.0001
HDL cholesterol, mean (SD), mmol/L	1.30 ± 0.39	1.30 ± 0.40	1.25 ± 0.36	1.44 ± 0.40	<0.0001
DM, *n* (%)					<0.0001
no	6550 (88.38)	4588 (89.07)	1444 (85.39)	518 (91.04)	
yes	861 (11.62)	563 (10.93)	247 (14.61)	51 (8.96)	
Hypertension, *n* (%)					<0.0001
no	4505 (60.79)	3310 (64.26)	870 (51.45)	325 (57.12)	
yes	2906 (39.21)	1841 (35.74)	821 (48.55)	244 (42.88)	
Dyslipidemia, *n* (%)					<0.0001
no	5027 (67.83)	3486 (67.68)	1078 (63.75)	463 (81.37)	
yes	2384 (32.17)	1665 (32.32)	613 (36.25)	106 (18.63)	

BMI, body mass index; SBP, systolic blood pressure; CRP, C-reactive protein; HbA1c, glycated hemoglobin; HDL-C, high-density lipoprotein cholesterol; DM, diabetes.

**Table 2:** Number and percentage of the changes in sarcopenia status.

Baseline	The Second Survey	Percentage (%)
non-sarcopenia	non-sarcopenia	2817 (81.3)
	possible sarcopenia	510 (14.7)
	sarcopenia	139 (4.0)
possible sarcopenia	non-sarcopenia	623 (58.6)
	possible sarcopenia	407 (38.3)
	sarcopenia	34 (3.2)
sarcopenia	non-sarcopenia	121 (41.0)
	possible sarcopenia	24 (8.1)
	sarcopenia	150 (50.8)

The time interval between baseline and the second survey was two years in the CHARLS.

**Table 3:** Association of changes in sarcopenia status with risks of incident depression.

		Crude Model	Model 1	Model 2	Model 3
Character	Events/*n*	HR (95%CI)	HR (95%CI)	HR (95%CI)	HR (95%CI)
Stable non-sarcopenia	1223/2817	reference	reference	reference	reference
Non-sarcopenia to possible sarcopenia/sarcopenia	325/649	1.33 (1.18, 1.50)	1.34 (1.18, 1.52)	1.3 (1.15, 1.47)	1.3 (1.14, 1.47)
Stable possible sarcopenia	216/407	reference	reference	reference	reference
Possible sarcopenia to non-sarcopenia	299/623	0.74 (0.62, 0.88)	0.73 (0.61, 0.87)	0.74 (0.62, 0.89)	0.75 (0.63, 0.91)
Possible sarcopenia to sarcopenia	16/34	1.16 (0.70, 1.93)	1.29 (0.77, 2.15)	1.24 (0.74, 2.07)	1.25 (0.74, 2.11)
Stable sarcopenia	75/150	reference	reference	reference	reference
Sarcopenia to non-sarcopenia/possible sarcopenia	63/145	0.57 (0.40, 0.80)	0.58 (0.40, 0.82)	0.62 (0.44, 0.89)	0.65 (0.45, 0.94)

Model 1 included adjustments for age and gender; Model 2 further adjusted for marriage, residence, and education level; and Model 3 additionally adjusted for drinking, smoking, physical activity function, BMI, SBP, CRP, HbA1c, TG, and HDL-C, as well as history of DM, hypertension and dyslipidemia. BMI, body mass index; SBP, systolic blood pressure; CRP, C-reactive protein; HbA1c, glycated hemoglobin; TG, Triglycerides; HDL-C, high-density lipoprotein cholesterol; DM, diabetes.

## Data Availability

The CHARLS datasets are publicly available at the project website: http://charls.pku.edu.cn. The data used in this specific study can be obtained from the corresponding author upon reasonable request.

## Supplementary Materials

The following supporting information can be downloaded at: https://www.mdpi.com/article/10.3390/jcm15114015/s1, Table S1A. The missing number and rate of covariates for baseline sarcopenia status analyses; Table S1B. The missing number and rate of covariates for changes in sarcopenia status analyses; Table S2. Baseline characteristics of participants for changes in sarcopenia status analyses; Table S3. Baseline characteristics of participants for baseline sarcopenia status analyses using non-imputed data; Table S4. Baseline characteristics of participants for changes in sarcopenia status analyses using non-imputed data; Table S5. Association of baseline sarcopenia status with risks of incident depression; Table S6. Subgroup of association of changes in non-sarcopenia group with risks of incident depression; Table S7. Subgroup of association of changes in possible-sarcopenia group with risks of incident depression; Table S8. Subgroup of association of changes in sarcopenia group with risks of incident depression; Table S9. Baseline characteristics of participants for changes in sarcopenia status in sensitivity analyses using data from the third survey; Table S10. Association of changes in sarcopenia status with risks of incident depression in sensitivity analyses using data from the third survey; Table S11. Baseline characteristics of participants for changes in sarcopenia status in sensitivity analyses adjusting antihypertensive and antidiabetic medications; Table S12. Association of changes in sarcopenia status with risks of incident depression in sensitivity analyses adjusting for antihypertensive and antidiabetic medications; Table S13. Association of changes in sarcopenia status with risks of incident depression in sensitivity analyses separating the possible sarcopenia/sarcopenia group from the non-sarcopenia/possible sarcopenia group; Table S14. Incidence rates of depression by baseline sarcopenia status; Table S15. Incidence rates of depression by changes in sarcopenia status; Supplementary Table S16. Comparison of baseline characteristics among the overall CHARLS population, the baseline analytical cohort, and the longitudinal transition cohort. Supplemental Methods; Supplemental Figure S1. Change trajectory of sarcopenia status, Supplemental Figure S2. Stable change trajectory of sarcopenia status when using the third survey, Supplementary Figure S3. Kaplan–Meier curves for participants with non-sarcopenia at baseline, comparing those who remained stable vs. those who progressed to possible sarcopenia or sarcopenia, Supplementary Figure S4. Kaplan–Meier curves for participants with possible sarcopenia at baseline, comparing stable possible sarcopenia, recovery to non-sarcopenia, and progression to sarcopenia. Supplementary Figure S5. Kaplan–Meier curves for participants with sarcopenia at baseline, comparing stable possible sarcopenia, recovery to non-sarcopenia, and progression to sarcopenia.

## Abbreviations

BMI	body mass index
CHARLS	China Health and Retirement Longitudinal Study
ASM	appendicular skeletal muscle mass
AWGS	Asian Working Group for Sarcopenia
DXA	dual X-ray absorptiometry
ASM/Ht2	height-adjusted muscle mass
SBP	systolic blood pressure
CRP	C-reactive protein
HbA1c	glycated hemoglobin
TG	Triglycerides
HDL-C	high-density lipoprotein cholesterol
DM	diabetes
CESD-10	10-item Center for Epidemiologic Studies Depression Scale
SD	standard deviation
IQR	interquartile range
HR	hazard ratio
CI	confidence intervals
HPA	hypothalamic–pituitary–adrenal
