# Supplementary material for: Changes in Sarcopenia and Incident Depression in Prospective Cohorts
Source: J Clin Med. 2026 May 22;15(11):4015. doi: 10.3390/jcm15114015 (PMC13257966; doi:10.3390/jcm15114015)

Supplemental Methods

Study designs of the CHARLS

The China Health and Retirement Longitudinal Study (CHARLS) was a prospective cohort study conducted in China. In wave 1, a nationally representative sample of 17,708 participants was recruited from 28 provinces in 2011 via multistage probability sampling. The primary aim of this study was to recruit participants aged ≥ 45 years, but some participants aged 40 to 44 years also attended the baseline survey. All 17,708 participants underwent face-to-face interviews by the trained staff using the standardized questionnaire to collect data on sociodemographic information, lifestyles, and health-related information. Among 17,708 participants, 13,978 participants conducted anthropometric measurements to collect data on height, weight, waist circumference, hip circumference, blood pressure, grip strength, and so on. In addition, 11,847 participants provided blood samples for the laboratory test. Biochemical indicators, including total cholesterol, high-density lipoprotein cholesterol, low-density lipoprotein cholesterol, glycated hemoglobin, fasting blood glucose, and C-reactive protein were measured by the blood test. The follow-up surveys were conducted in 2013 (wave 2), 2015 (wave 3), and 2018 (wave 4) with questionnaire interviews and anthropometric measurements. Blood samples were collected in 2015 again, and biochemical indicators were measured by the blood test.

Ascertainment of depression

The depression of the respondents in our study was assessed using the 10-item Center for Epidemiologic Studies Depression Scale (CESD-10)(1). The scale evaluates the respondent's psychological situation in the past week. It comprises questions in 10 aspects: "I was bothered by things that do not usually bother me," "I had trouble keeping my mind on what I was doing," "I felt depressed," "I felt everything I did was an effort," "I felt hopeful about the future," "I felt fearful," "My sleep was restless," "I was happy," "I felt lonely," and "I could not get going." The choices were "Rarely or none of the time (<1day)," "Some or a little of the time (1–2days)," "Occasionally or a moderate amount of the time (3–4days)," and "most of the time (5–7days)," which were scored 0~3, and two questions reflecting positive sentiment (I felt hopeful about the future, I was happy) were achieved in reverse (3~0). The final score was calculated by adding the scores of the ten questions. The total scores ranged

from 0 to 30, and the participants were considered with depression when the total score was ≥10 (2-4).

Multiple imputation

The missing data of covariates were imputed using the multiple imputation with chained equation(5). In the CHARLS, we only imputed the covariates in which the missing rates were less than 80% recommended by previous studies(6, 7).All eligible covariates were imputed using one imputation model which included the age, sex, education, marital status, smoking status, drinking status, body mass index, systolic blood pressure, glycated hemoglobin, high-density lipoprotein cholesterol, C-reactive protein, antihypertensive drug, and antidiabetic drug. In each cohort, we performed 5 imputations and generated 5 imputed datasets. Effect estimates were computed separately for each of the 5 datasets, and then combined according to Rubin's rules(5).The multiple imputation was conducted using the R package "mice".

Reference

1. Andresen EM, Malmgren JA, Carter WB, Patrick DL. Screening for depression in well older adults: evaluation of a short form of the CES-D (Center for Epidemiologic Studies Depression Scale). Am J Prev Med. 1994;10(2):77-84.
2. Rong H, Lai X, Jing R, Wang X, Fang H, Mahmoudi E. Association of Sensory Impairments With Cognitive Decline and Depression Among Older Adults in China. JAMA Netw Open. 2020;3(9):e2014186.
3. Du X, Liao J, Ye Q, Wu H. Multidimensional Internet Use, Social Participation, and Depression Among Middle-Aged and Elderly Chinese Individuals: Nationwide Cross-Sectional Study. J Med Internet Res. 2023;25:e44514.
4. Zhou L, Ma X, Wang W. Relationship between Cognitive Performance and Depressive Symptoms in Chinese Older Adults: The China Health and Retirement Longitudinal Study (CHARLS). J Affect Disord. 2021;281:454-8.
5. White IR, Royston P, Wood AM. Multiple imputation using chained equations: Issues and guidance for practice. Stat Med. 2011;30(4):377-99.
6. van Es N, Takada T, Kraaijpoel N, Klok FA, Stals MAM, Büller HR, et al. Diagnostic management of acute pulmonary embolism: a prediction model based on a patient data meta-analysis. Eur Heart J. 2023;44(32):3073-81.
7. Madley-Dowd P, Hughes R, Tilling K, Heron J. The proportion of missing data should not be used to guide decisions on multiple imputation. J Clin Epidemiol. 2019;110:63-73.

Supplemental Figure Legends

Supplemental Figure S1. Change trajectory of sarcopenia status

In main analyses, changes in sarcopenia status were assessed based on the sarcopenia status at baseline and the second survey which was conducted two years after the baseline.

Because the sample sizes of non-sarcopenia to sarcopenia and sarcopenia to non-sarcopenia trajectories were small, we integrated these two trajectories into non-sarcopenia to possible sarcopenia /sarcopenia and sarcopenia to non-sarcopenia/possible sarcopenia.

Figure S1

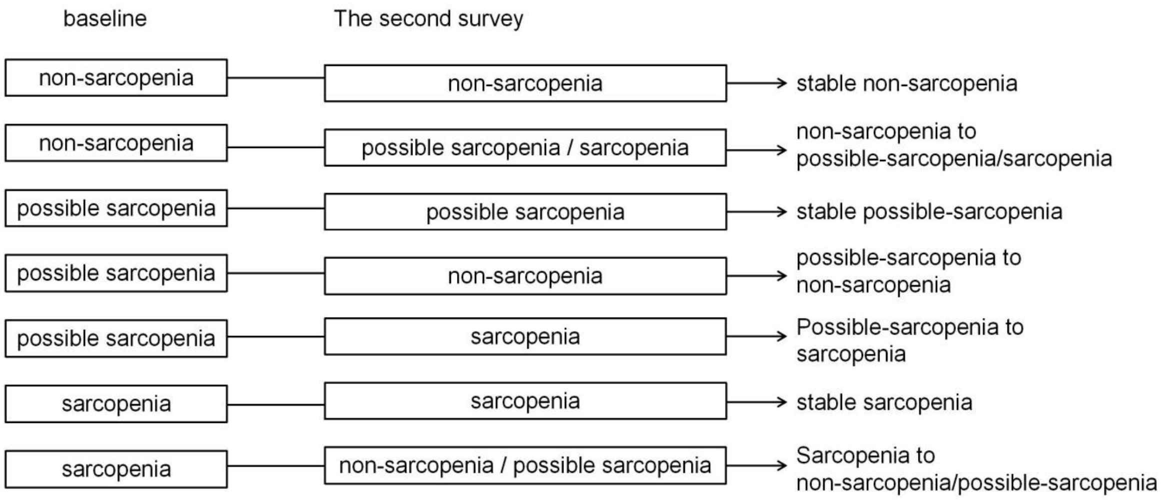

Supplemental Figure S2. Stable change trajectory of sarcopenia status when using the third survey

In sensitivity analyses, to reduce the bias from potentially unstable changes in sarcopenia status, we used the third survey (wave 3 of CHARLS) to ensure the stability of sarcopenia changes.

Unstable changes in sarcopenia status meant that sarcopenia status changed again at the third survey, such as participants who changed from possible sarcopenia to non-sarcopenia from baseline to the second survey, but became possible sarcopenia again at the third survey. Participants with unstable changes in sarcopenia status were excluded from this analysis.

Stable changes in sarcopenia status meant that sarcopenia status remained stable at the third

survey, such as participants who changed from possible sarcopenia to non-sarcopenia from baseline to the second survey, and remained non-sarcopenia at the third survey. Participants with stable changes in sarcopenia status were included in this analysis. The sample sizes for this analysis were 3816 in the study

Figure S2

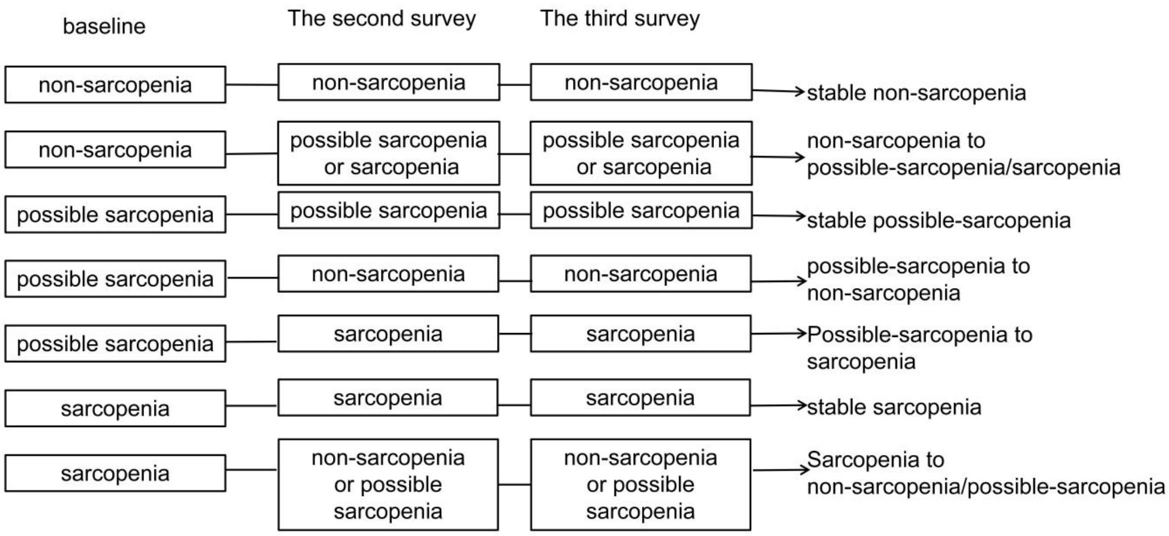

Supplementary Figure S3. Kaplan-Meier curves for participants with non-sarcopenia at baseline, comparing those who remained stable vs. those who progressed to possible sarcopenia or sarcopenia.

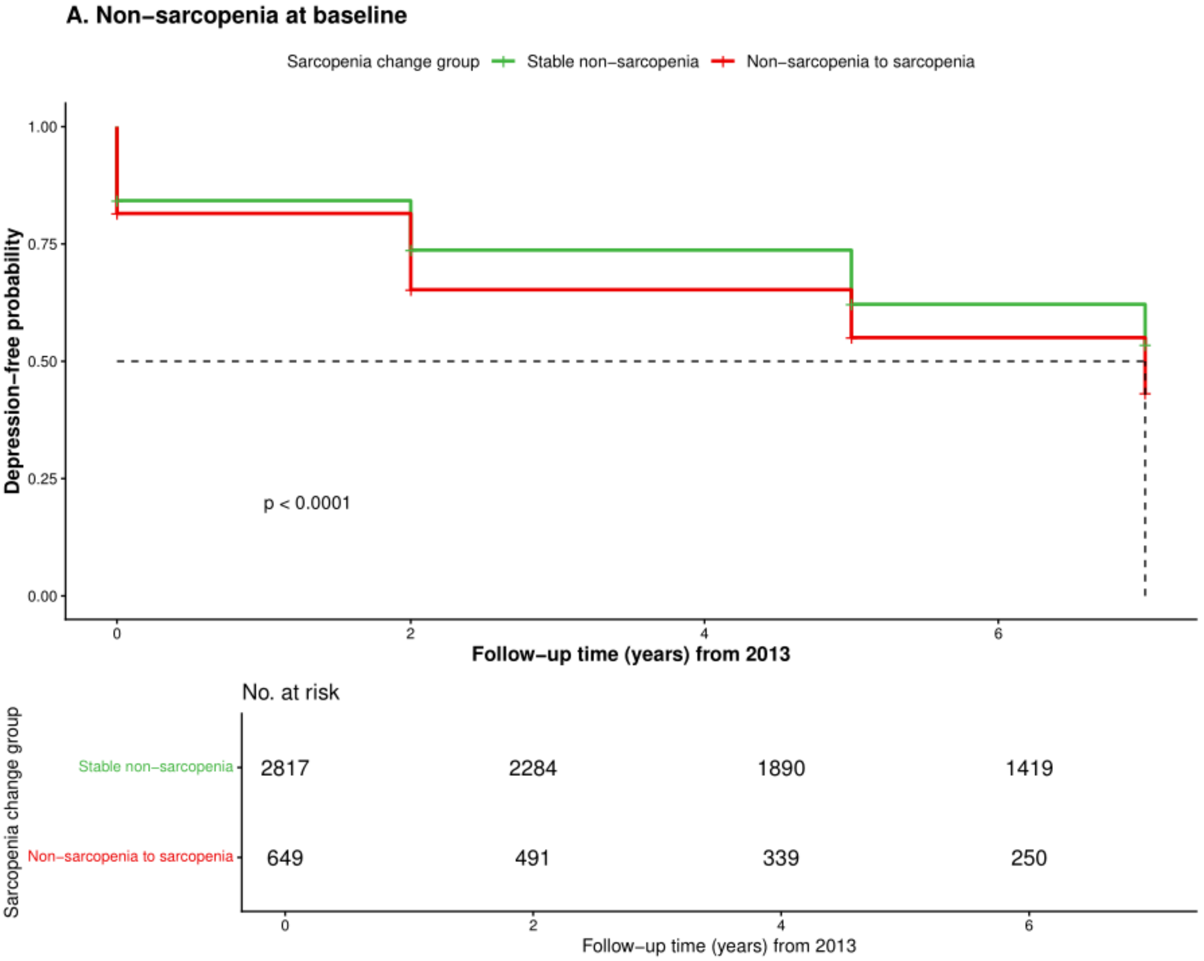

Supplementary Figure S4. Kaplan-Meier curves for participants with possible sarcopenia at baseline, comparing stable possible sarcopenia, recovery to non-sarcopenia, and progression to sarcopenia.

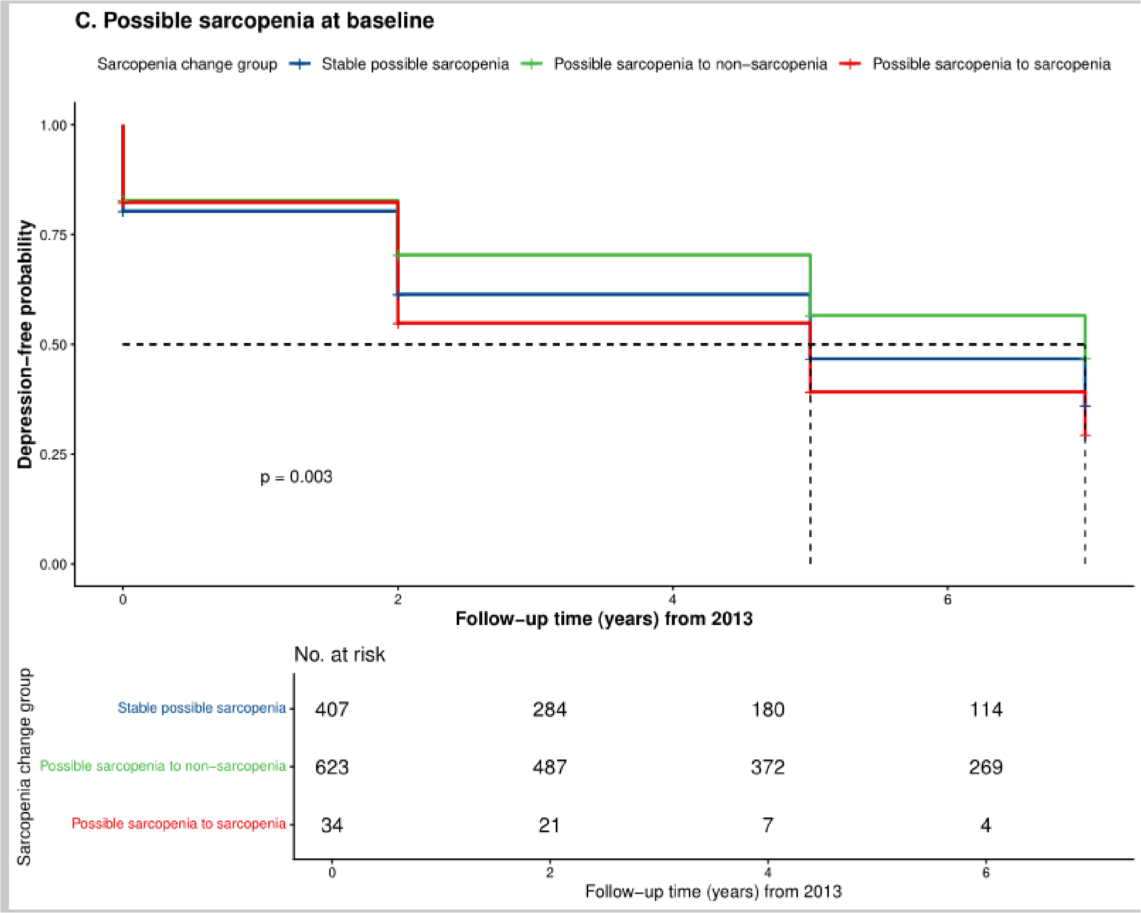

Supplementary Figure S5. Kaplan-Meier curves for participants with sarcopenia at baseline, comparing stable sarcopenia vs. recovery to non-sarcopenia or possible sarcopenia.

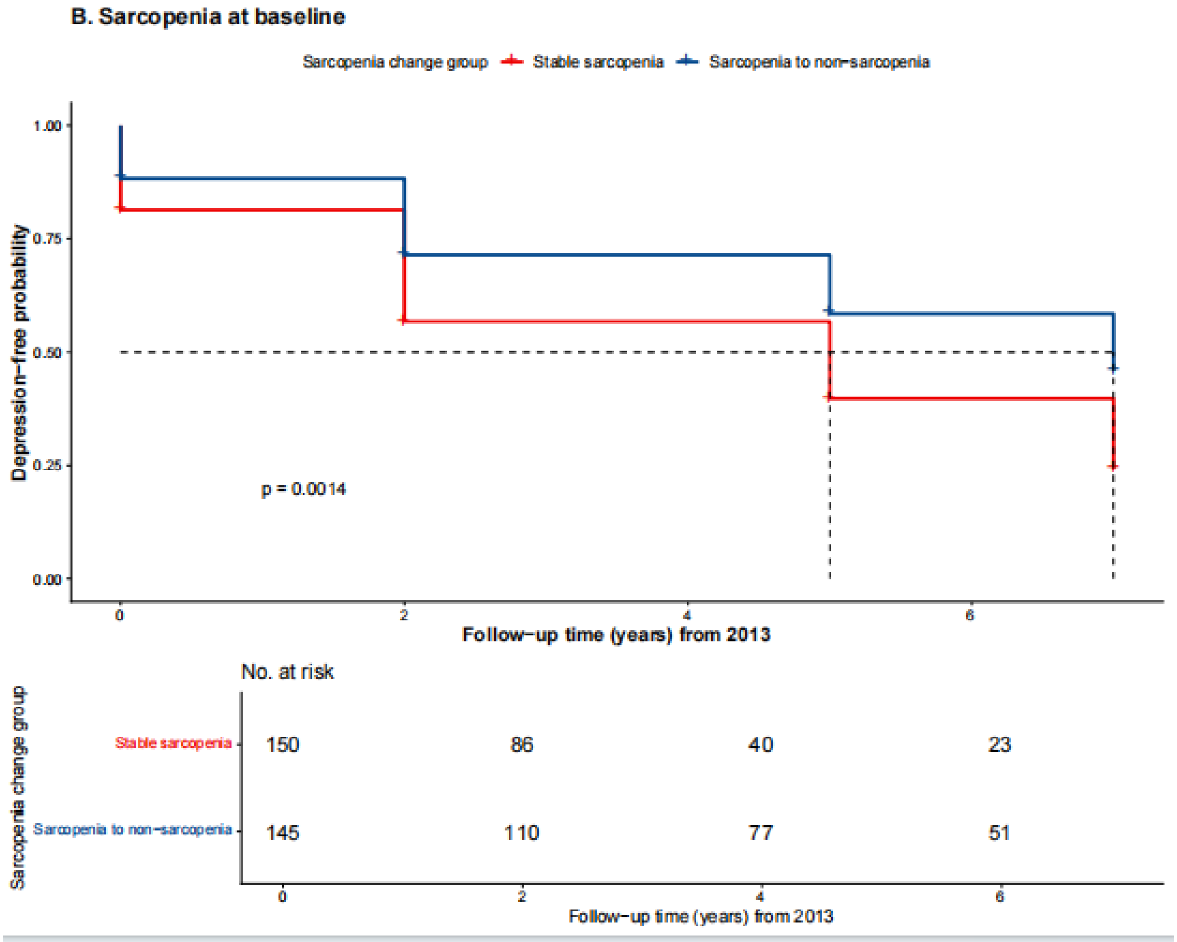

Supplement: Supplementary file 1 [file jcm-15-04015-s001.zip › Supplemental Methods.pdf]
